# Involvement of nitric oxide in 5-HT_3_ receptor agonist-induced fluid accumulation in jejunum and colon of anesthetized rats

**DOI:** 10.4103/0253-7613.58511

**Published:** 2009-10

**Authors:** B. Veeresh, Basanagouda M. Patil, S.V. Veeresh Babu, Neelakanth M. Jeedi, Banappa S. Unger

**Affiliations:** Department of Pharmacology, K.L.E.Ss College of Pharmacy, J.N.M.C. Campus, Nehru Nagar, Belgaum - 590 010, India; 1 Vidyanagar, Hubli - 580 031, Karnataka, India

**Keywords:** D-arginine, L-arginine, Nω-nitro-L-arginine, 1-phenylbiguanide

## Abstract

**Objectives:**

The aim of the present study was to investigate the involvement of nitric oxide in 5-HT_3_ receptor agonist-induced fluid accumulation in jejunum and colon of anesthetized rats.

**Materials and Methods:**

Fluid movement in jejunum and colon were determined simultaneously in the same rat, by modifying the Beubler method. Nω-nitro-L-arginine (L-NNA, 20 mg/kg, s.c) alone and in combination with L-arginine (L-Arg, 150 mg/kg s.c) or D-arginine (D-Arg, 150 mg/kg, s.c) were administered 30 min before administration of 1-PBG (18.5 μg/kg, i.v).

**Results:**

Intravenous administration of 1-phenylbiguanide (1-PBG) induced a net secretion of fluid in both jejunum and colon. 1-PBG had a more prominent secretory effect in the colon, causing a three-fold increase in volume of fluid secreted/g of colon than in the jejunum. Pretreatment with (L-NNA) prevented the 1-PBG-induced fluid accumulation in both jejunum and colon. The inhibitory effect of L-NNA on 1-PBG-induced fluid accumulation was reversed by L-Arg but not by D-Arg.

**Conclusion:**

These results provide evidence that nitric oxide plays an important role in 5-HT_3_ receptor agonist-induced fluid accumulation in jejunum and colon of anesthetized rats.

## Introduction

5-Hydroxytryptamine_3_ (5-HT_3_) receptors are widely distributed in the enteric nervous system within the gastro-intestinal tract.[1] They are responsible for neurally mediated 5-hydroxytryptamine (5-HT)-induced secretion in rat colon[2] and intestine.[3] Nitric oxide (NO) resulting from L-arginine by the action of stereo-specific enzyme nitric oxide (NO) synthase in the gut mediated fluid secretion in intestine[4] and colon.[5] Further, NO synthase inhibitors prevented diarrhea and intestinal fluid and electrolyte secretion induced by laxatives.[6,7] Involvement of NO in the 5-HT-induced diarrhea and intestinal secretion has been reported.[8,9] In these studies, NO synthase inhibitors caused partial inhibition of 5-HT-induced secretion and it was proposed that NO may play role in the secretory response to 5-HT which is mediated through neural mechanisms.[8] These reports provide an opportunity for further exploration of the involvement of NO in the intestinal secretory response to 5-HT_3_ receptors. The aim of the present study was to investigate the involvement of nitric oxide in 1-phenylbiguanide (1-PBG), a selective 5-HT_3_ agonist-induced fluid secretion[2,3] in the jejunum and colon of anesthetized rats, using NO synthase inhibitor Nω-nitro-L-arginine (L-NNA) and L-arginine, precursor of NO synthase.

## Materials and Methods

### Materials

1-Phenylbiguanide (Aldrich, Gillingham, dorset, UK), L-NNA (Fluka Chemie AGCH-9470 Buchs, Switzerland), L-arginine hydrochloride and D-arginine hydrochloride (Sigma Chemical Company St Louis, USA), and atropine sulphate (Hi-Media, Bombay, India) were purchased for the study. Ondansetron hydrochloride dehydrate (Cipla limited company, Bombay, India) was procured as a gift sample. All drugs were dissolved freshly in isotonic saline. All chemicals used for the Tyrode and other solutions were of extra pure quality available from commercial sources.

### Animals

Wister rats of either sex (150-180 g) obtained from the National Institute of Nutrition, Hyderabad, India, were used after 1 week of acclimatization (temperature 35 ± 2°C). Food was withheld 18 h before experiment, but free access to drinking water was allowed and each rat was placed in a separate cage. The experimental design and procedures were approved by the Institutional Ethical Committee for Animal Care and Use at the K.L.E.S. College of Pharmacy, Belgaum.

### Measurement of fluid movement in jejunum and colon in vivo

Fluid movements in jejunum and colon were determined simultaneously in the same rat, by modifying the Beubler method.[10] The rats were anesthetized with sodium pentobarbitone (60 mg/kg, i.p). The abdomen was opened and a polythene catheter (No. 8) was placed in the jejunum, about 5 cm distal to the flexuraduodenojejunalis and fixed by ligation, a second ligature was placed approximately 20 cm distally. Similarly, the colon was cannulated proximally about 5 cm distal to the ileo-caecal junction with polythene catheter (No. 8) and a second ligature was placed at the distal end of the colon.

The jejunum and colon were rinsed carefully with 10 ml and 20 ml of warm sterile saline solution (37°C), respectively, to remove the contents followed by blowing air with the help of syringe. The distal ends of both parts were closed by ligation. One hour after the preparation, 2 ml of pre-warmed (37°C) Tyrode solution (composition g/l: NaCl-8.0, KCl-0.2, CaCl_2_-0.2, MgCl_2_-0.1, NaHCO_3_-0.1, NaH_2_PO_4_-0.05, D-glucose 1.0) was instilled in both jejunum and colon and catheters were withdrawn before tying of the proximal end. 1-PBG or saline was administered through femoral vein and washed with 0.2 ml saline, just before instillation of Tyrode solution.

After 30 min, the jejunum and colon were removed from the animal and the volume of the fluid content was noted. The animals were killed by an overdose of pentobarbitone. The empty loop was weighed and fluid transport was taken as the difference between the initial and final volume in the loop and expressed as μl/g wet weight/30 min. A negative value denotes absorption and a positive value denotes secretion. Testing agents either alone or in combination were administered s.c. 30 min before i.v. of 1-PBG.

### Data and statistical analysis

All data were expressed as mean ± SEM. Unpaired Student's ‘*t*’ test was used to determine the statistical significance of differences between groups. *P* < 0.05 was taken as statistically significant.

## Results

**Figure 1:**
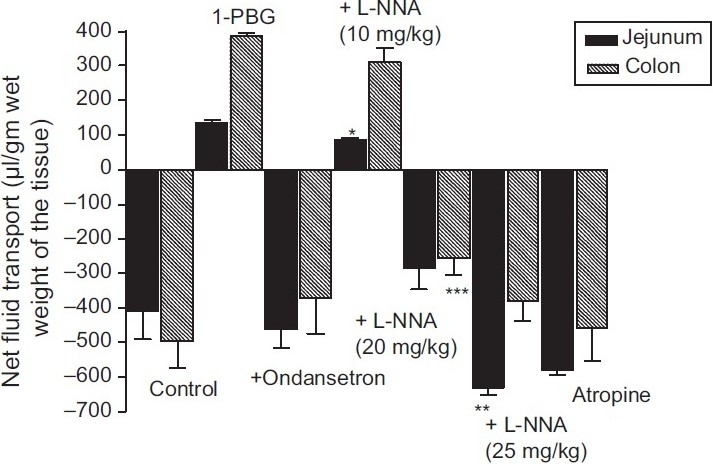
Effect of ondansetron, L-NNA and atropine on 1-PBG-induced intraluminal fluid transport. Results are expressed as mean ± S.E.M (n = 6). A negative value represents net absorption and positive value represents net secretion; **P < 0.02; ***P < 0.01 vs. control; *P < 0.001 vs. 1-PBG

As shown in Figure 1, under control conditions net absorption of fluid occurred in both the jejunum and colon. Intravenous administration of 1-PBG (18.5 μg/kg) induced a net secretion of fluid in both jejunum and colon. 1-PBG had a more prominent secretory effect in the colon, causing a three-fold increase in volume of fluid secreted/g of colon than in the jejunum. Pretreatment with ondansetron (150 mg/kg), a selective 5-HT_3_ antagonist, reversed 1-PBG-induced secretion to absorption in the both regions of the gut. Similarly, pretreatment with atropine (5 mg/kg) reversed 1-PBG-induced secretion to absorption in both the jejunum and colon. This dose of 1-PBG was used to examine the effects of L-NNA.

Pretreatment with NOS inhibitor L-NNA (10, 20, 25 mg/ kg) dose dependently modified 1-PBG-induced fluid secretion in both the jejunum and colon. In the jejunum, the dose of L-NNA in the range of 10-20 mg/kg inhibited the 1-PBG-induced fluid secretion, and increasing the dose to 25 mg/kg enhanced the fluid absorption significantly more than control levels (*P* < 0.02). While in the colon the dose of L-NNA in the range 10-20 mg/kg inhibited the 1-PBG-induced fluid secretion in a dose-related manner and 25 mg/kg dose returned net absorption of fluid near to control levels [Figure 1].

**Figure 2:**
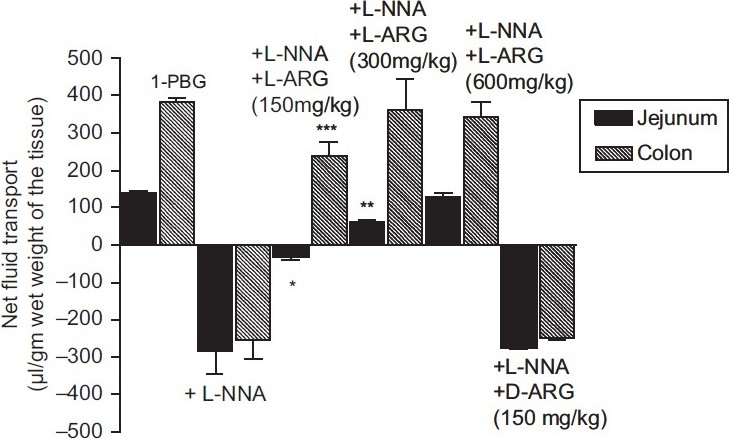
Effect of combined administration of L-NNA and L-arginine or D-arginine on 1-PBG-induced intraluminal fluid transport. Results are expressed as mean ± S.E.M. (n = 6). A negative value represents net absorption and positive value represents net secretion. ***P < 0.01, **P < 0.001 vs. 1-PBG; *P < 0.01 vs. 1-PBG + L-NNA

L-arginine (150-600 mg/kg) reversed the effect of L-NNA (20 mg/kg) on 1-PBG-induced fluid accumulation in the gut in a dose-related fashion. A 600 mg/kg dose of L-arginine and a 300 mg/kg dose of L-arginine abolished completely the inhibitory effect of L-NNA in jejunum and colon, respectively. In contrast, D-arginine (300 mg/kg) did not alter the effects of L-NNA in rats treated with 1-PBG [Figure 2].

## Discussion

It has been reported that 1-PBG a selective 5-HT_3_ agonist induces secretion in rat intestine and colon.[3,2] We obtained reproducible fluid accumulation in jejunum and colon after intravenous administration of 1-PBG which were abolished by ondansetron a selective 5-HT_3_ receptor antagonist. Secretory response of 1-PBG was about three-fold more in colon compared to jejunum, and this difference in the effect of 1-PBG was not surprising since 5-HT_3_ receptor contribution to the stimulation of electrogenic chloride secretion by 5-HT has been reported to be more in colon compared to small intestine.[11]

The involvement of NO in the secretory response to 5-HT_3_ receptor stimulation was examined in the present study, in which 1-PBG response was tested after pretreatment with NOS inhibitor L-NNA subcutaneously. We observed that L-NNA abolished the secretory response of 1-PBG in both jejunum and colon and the effect of L-NNA was dose dependent. Further, the NO synthase substrate, L-arginine reversed the inhibitory effect of L-NNA on secretory response induced by 1-PBG. This effect was enantiomer specific because D-arginine did not show any effect on L-NNA action. The dose of L-arginine that reverses the NO synthase inhibitor effect can be 3-100 fold higher than that of the NO synthase inhibitor, depending on the tissue and species studied.[12] In the present study, the dose of L-arginine required was 15 and 30 fold higher than L-NNA in colon as well as jejunum. Thus, the results suggest that 5-HT_3_ receptor evoked fluid secretion in the jejunum and colon of rat involves the L-arginine NO pathway.

There is general agreement that 5-HT_3_ receptor in the intestinal tract are located on enteric sensory neurons and activate a cholinergic mechanism to stimulate secretion.[13,14] Our results are in agreement with these reports since atropine a muscarnic receptor antagonist abolished the secretory response of 1-PBG which acts solely via neural mechanisms.[15] In the gastro-intestinal tract, NOS has been localized in myentric and submucosal neurons, subepithelial compartment, and lamina propria. Further, several studies have demonstrated NO as a secretagoge in the jejunum and colon in castor oil and bisacodyl-induced secretory states.[4–7] Based on these reports and our observation, it appears that the activation of 5-HT_3_ receptors in the gut may activate neurons that generate NO. The generated NO in turn could activate secretory mechanisms. Since both atropine and L-NNA could abolish 1-PBG-induced secretory effects independently, it is likely that release of neurotransmitter, acetylcholine, and NO may be synaptically coupled.

It is therefore concluded that NO may play a role in the secretory response of rat jejunum and colon to 5-HT_3_ receptor stimulation. Secretory response of 5-HT_3_ receptor stimulation is more prominent in colon than in jejunum of rat.
